# Primary Cardiac Lymphoma Presenting with Thrombocytopenia, Right Heart Failure, and Cardiogenic Shock

**DOI:** 10.1155/2023/5501131

**Published:** 2023-01-05

**Authors:** Samantha Kurniawan, Gita Mathur, Yvonne Bogun, Giselle Kidson-Gerber

**Affiliations:** ^1^Department of Haematology, Prince of Wales Hospital, Randwick, NSW 2031, Australia; ^2^Department of Cardiology, Prince of Wales Hospital, Randwick, NSW 2031, Australia; ^3^Department of Pathology, Prince of Wales Hospital, Randwick, NSW 2031, Australia; ^4^University of NSW, Randwick, NSW 2031, Australia

## Abstract

Primary cardiac lymphoma (PCL) is a rare, potentially fatal subtype of non-Hodgkin's lymphoma. Thrombocytopenia has also infrequently been reported in association with other primary cardiac tumours and can add substantial morbidity to an already life-threatening diagnosis if present. We report a rare case of a 70-year-old man who presented with thrombocytopenia (91 × 10^9^/L) and progressive right heart failure. Transthoracic echocardiogram revealed a large 8 × 4 cm right atrial mass with severe tricuspid obstruction, confirmed as PCL on subsequent endomyocardial biopsy and immunohistochemistry. He deteriorated into cardiogenic shock precipitated by atrial fibrillation, with worsening thrombocytopenia (18 × 10^9^/L) in the setting of ischaemic hepatitis. The patient stabilised with initiation of high dose steroids prior to tissue diagnosis and platelet counts normalised following chemotherapy. This case demonstrates the importance of considering PCL as a diagnosis and preemptive initiation of high dose steroids to improve outcomes in PCL associated with cardiogenic shock. This case also elucidates a potential pathophysiological association between PCL and thrombocytopenia.

## 1. Introduction

Secondary cardiac involvement by disseminated lymphoma is common, observed in up to 24% of patients in an autopsy series [1]. In contrast, primary cardiac lymphoma (PCL), defined as extranodal lymphoma involving only the heart or pericardium, represents a rare subtype of non-Hodgkin's lymphoma associated with poor outcomes [2]. Patients with PCL typically present with dyspnoea and constitutional symptoms such as weight loss and anorexia [2]. Thrombocytopenia has rarely been described in association with PCL [3] and other primary cardiac tumours [4–8] and can add substantial morbidity to the diagnosis if present. We report to our knowledge, the second case of primary cardiac lymphoma presenting with marked thrombocytopenia which subsequently resolved with chemotherapy.

## 2. Case Presentation

**Figure 1:**
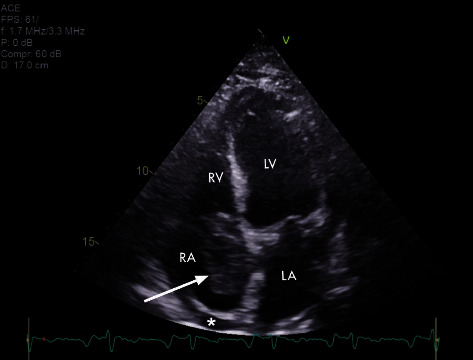
Transthoracic echocardiogram. Apical four chamber view demonstrating a large right atrial mass (arrow) causing tricuspid valve obstruction with associated pericardial effusion (asterisk). Abbreviations: RA (right atrium), RV (right ventricle), LV (left ventricle), and LA (left atrium).

A 70-year-old man presented with subacute progressive exertional dyspnoea, anorexia, and 7 kg weight loss over 6 months at a peripheral hospital on a background history of allergic bronchopulmonary aspergillosis and Peyronie's disease. He was haemodynamically stable, in right heart failure with raised jugular venous pressure, ascites, and bilateral lower limb oedema. His platelet count was 91 × 10^9^/L, compared to 255 × 10^9^/L three months prior. Blood film revealed normal platelet morphology with no clumping or red cell fragments. CT imaging demonstrated an 8 × 4 cm right atrial mass, moderate pericardial and left pleural effusion, ascites, and bilateral lower lobe pulmonary emboli. A heparin infusion was commenced. The transthoracic echocardiogram (TTE) confirmed a large right atrial mass extending into the right ventricle and origin of the inferior vena cava resulting in severe tricuspid obstruction and RV dilatation, with an associated 1.9 cm pericardial effusion without signs of tamponade (Figure 1). Percutaneous pericardiocentesis was attempted unsuccessfully due to difficult body habitus with significant ascites. Electrocardiography revealed a new right bundle branch block with first degree heart block.

**Figure 2:**
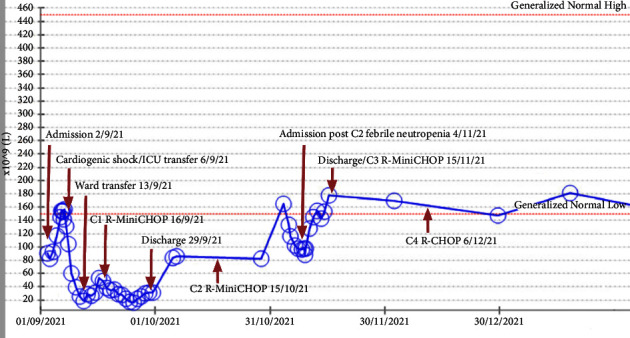
Trend of platelet count in units 10^9^/L throughout treatment.

On day 4 of admission, he decompensated into severe cardiogenic shock precipitated by rapid atrial fibrillation requiring amiodarone to restore sinus rhythm and urgent transfer to our hospital's intensive care for inotropic support. This was further complicated by the development of ischaemic hepatitis with an associated coagulopathy (PT 50 s, INR 4.4, APTT 34.5 s, fibrinogen 3.0 g/L, and D-dimer 3.49 mg/L) and worsening thrombocytopenia to a nadir of 18 × 10^9^/L on day 10 of admission, clinically manifesting with recurrent epistaxis and haematuria (Figure 2). The antiheparin-PF4 IgG chemiluminescent immunoassay was negative.

**Figure 3:**
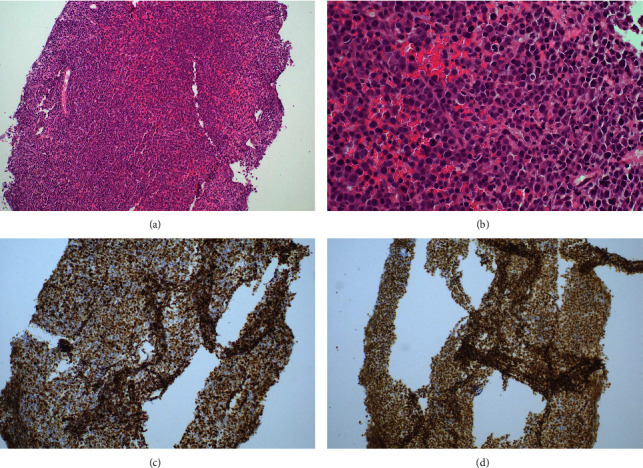
Endomyocardial biopsy. Hematoxylin and eosin staining ((a) x10 magnification and (b) high power view) demonstrates atypical large lymphocytes confirmed as B-cells on Pax5 immunohistochemistry (c). Ki67 stain (d) demonstrates a high proliferation rate.

**Figure 4:**
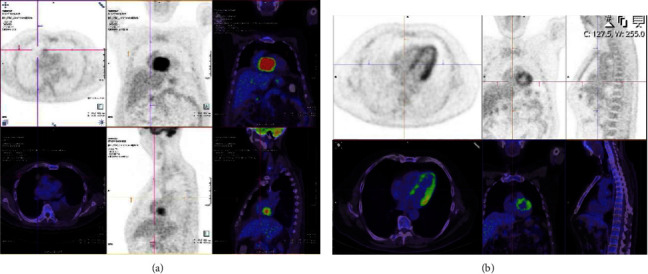
Initial PET (a) demonstrates focal intense FDG uptake corresponding to a right sided cardiac mass (SUVmax 20.6), no longer evident on progress PET (b) in keeping with complete metabolic response. There is moderate physiological uptake in the left ventricular myocardium seen in (b).

He was empirically commenced on intravenous hydrocortisone hoping it would improve his guarded prognosis as lymphoma was considered the only significantly treatable differential for his right atrial mass. Endomyocardial biopsy of this mass confirmed large atypical lymphocytes staining positive for CD20, bcl-2, bcl-6, and c-myc consistent with an aggressive B-cell lymphoma (Figure 3). Flow cytometry confirmed the abnormal B-cell population with CD10 neg, CD5+, CD 19+, CD20+, CD23 neg, CD79b+, FMC7 neg, CD200+, and monoclonal lambda surface immunoglobulin light chains. Positron emission tomography (PET) revealed focal intense FDG uptake in the right atrial mass with no evidence of any other metabolically active nodal or extranodal disease, consistent with a diagnosis of primary cardiac lymphoma (Figure 4). He was weaned off inotropic support and transferred out of ICU at day 11 of admission. He was treated with three cycles of R-MiniCHOP (dose-adjusted for liver dysfunction), three cycles of R-CHOP, and two further cycles of rituximab. His platelet count normalised to 154 × 10^9^/L after two cycles of half-dose R-CHOP and has remained normal (Figure 2). Posttreatment PET demonstrated complete metabolic response.

## 3. Discussion

Primary cardiac lymphoma (PCL) is a rare subtype of non-Hodgkin's lymphoma. It typically presents with a male predominance in the 6th decade with dyspnoea, chest pain, and constitutional complaints [2]. Patients can encounter complications such as atrial arrhythmias, atrioventricular blocks, and significant haemodynamic compromise as exemplified in our case [2, 9]. The prognosis for untreated PCL is exceedingly poor with survival of less than 1 month; however, median survival is extended to 45.4 months with appropriate management, usually R-CHOP chemotherapy [10]. In our case, early consideration of PCL with empirical high dose steroid therapy prior to tissue diagnosis likely made a significant improvement to our patient's prognosis in the setting of cardiogenic shock.

Another novel observation in our case was the prominent finding of isolated thrombocytopenia (91 × 10^9^/L) on presentation. Thrombocytopenia is broadly caused by increased peripheral platelet destruction or reduced platelet production in the bone marrow. Our patient had no previous history or risk factors for chronic liver disease and no splenomegaly on CT. He had normal HBV, HCV, and HIV serologies, in addition to normal B12 and folate. Lymphomatous infiltration of the bone marrow as a cause of his thrombocytopenia was less likely given a lack of FDG marrow uptake on PET and otherwise normal Hb 150 g/L, WCC 6.4 × 10^9^/L and film. A negative antiheparin-PF4 IgG chemiluminescent immunoassay excluded heparin-induced thrombocytopenia. Notably, his platelet count significantly deteriorated further to a nadir of 18 × 10^9^/L in the setting of ischaemic hepatitis from PCL-induced cardiogenic shock. Following resolution of ischaemic hepatitis, his thrombocytopenia partially recovered to 80 × 10^9^/L, before normalising entirely following two cycles of chemotherapy.

Cases of thrombocytopenia resolving immediately with surgical resection of primary cardiac tumours such as atrial myxoma and sarcoma have been described in the literature [4–8]. Proposed mechanisms of thrombocytopenia associated with cardiac tumours have included mechanical shearing via turbulent obstructed flow through the heart and platelet consumption by microthrombi formation within the tumour itself [4, 6]. We speculate similar mechanisms in our case given the size of our patient's tumour with severe tricuspid obstruction and with thrombocytopenia resolution following tumour reduction by chemotherapy. Another possible mechanism is thrombocytopenia resulting from immune thrombocytopenia purpura (ITP) which has been described in relation to non-Hodgkin's lymphoma (NHL) [11]. In the only other known case of thrombocytopenia associated with primary cardiac lymphoma, ITP was thought to be the cause and developed following 3 cycles of chemotherapy with partial remission attained with prednisone [3].

In conclusion, we describe the second known case of primary cardiac lymphoma associated with thrombocytopenia. We speculate this is due to tumour-induced mechanical shear and platelet consumption; however, regardless of the mechanism, our case and previous literature suggest that sustained resolution of thrombocytopenia can be achieved with tumour-directed therapy. This case demonstrates the importance of considering PCL as a diagnosis and a possible role for preemptive initiation of high dose steroids to improve outcomes in PCL associated with cardiogenic shock.

## Data Availability

The data used to support the findings of this study can be obtained from the corresponding author upon request.
